# How do the neurocognitive profiles of FASD and complex trauma compare in the pediatric population?: A scoping review protocol

**DOI:** 10.1371/journal.pone.0328845

**Published:** 2025-08-05

**Authors:** Rishi Gupta, Colleen Pawliuk, Denise Somuah-Asamoah, Katelynn E. Boerner, Jennifer Engle, Sarah Hutchison, Gurpreet K. Salh

**Affiliations:** 1 Sunny Hill Health Center, BC Children’s Hospital, Vancouver, British Columbia, Canada; 2 Faculty of Medicine, University of British Columbia, Vancouver, British Columbia, Canada; 3 Department of Pediatrics, University of British Columbia, Vancouver, British Columbia, Canada; UFRN: Universidade Federal do Rio Grande do Norte, BRAZIL

## Abstract

**Objective:**

The aim of this scoping review is to compare the neurocognitive profiles of children and youth diagnosed with Fetal Alcohol Spectrum Disorder (FASD) to those of children and youth diagnosed with complex trauma.

**Introduction:**

The neurocognitive challenges resulting from prenatal alcohol exposure (PAE) have been defined by the FASD diagnosis. Complex trauma exposure, in the form of adverse childhood experiences (ACEs), is associated with similar neurocognitive deficits. Recent evidence suggests ACE exposures to be higher in individuals with FASD as compared to non-FASD controls which warrants a closer look at the overlap between the neurocognitive deficits associated with FASD and complex trauma. A more robust understanding comparing the neurocognitive profiles of FASD and complex trauma can guide assessment, diagnosis, and interventions to support the long-term management of youth with cognitive impairments.

**Inclusion criteria:**

The review will assess various studies that evaluate the neurocognitive profiles of FASD and complex trauma amongst the pediatric population (aged 0–18). Complex trauma will be defined as multiple interpersonal traumatic events that happen in childhood. The context of this review is pediatric patients diagnosed with FASD or exposed to complex trauma.

**Methods:**

We will search MEDLINE (Ovid), Embase (Ovid), PsycINFO (EBSCO), Scopus and Google Scholar. Additionally, will conduct backwards and forwards citation searching of all included sources. Title and abstract screening and full-text review will be performed by at least two independent reviewers. Data extraction will be performed using a tool developed for this review. The results will be presented in a narrative and tabular format.

## Introduction

It has been known for decades that prenatal alcohol exposure (PAE) during gestation complicates the health of the mother and causes a myriad of developmental, neurocognitive, and physical impairments in the developing fetus [1–5]. In 2019, it was estimated that approximately 10–15% of pregnant women consumed alcohol in the United States and Canada [5]. Fetal alcohol spectrum disorder (FASD) is a diagnostic term used to describe the cognitive, developmental, and physical effects of PAE [6]. The teratogenic effects of alcohol on the developing fetus are influenced by many factors including the amount consumed, the gestational time at which alcohol consumption took place, and maternal factors including age and nutrition [3]. FASD diagnosis involves a clinician following a detailed algorithm to assess for sentinel facial features (although not necessary for a FASD diagnosis), central nervous system impairments, and microcephaly, depending on the age of the child [6]. The long-term effects of PAE have been well documented and include deficits in physical, intellectual, and social development as well as increased risk of suicidality [7]. As of 2019, the prevalence of FASD in Canada amongst children aged 1–17 was 0.1% or 1 per 1000 [8]. Given its impact on the lives of children and adolescents with FASD, it is imperative that supports and systems are in place to provide appropriate care.

Similar to PAE, exposure to one or more postnatal adverse childhood experiences (ACEs) has been shown to increase the relative risk of neurodevelopmental diagnoses in youth [9–11]. ACEs are traumatic events that occur at a young age and include neglect, living with household members who misused substances, as well as any form of psychological, physical or sexual abuse [12]. Exposure to 4 or more ACEs has been shown to increase an individual’s health risk for substance misuse, depression, and alcoholism by 4–12 fold [12]. Exposure to multiple ACEs in childhood is further associated with physical inactivity, smoking, chronic health conditions including heart and respiratory disease, as well as impaired mental health into adulthood [13]. We used the term ‘complex trauma’ as our variable of interest to capture exposure to multiple, interpersonal traumatic events that occurred in childhood or adolescence [14–17].

Exposure to PAE or complex trauma can result in long-term neurocognitive deficits in the developing fetus [18–20]. Given its teratogenic nature, PAE affects fetal brain development in various regions including the corpus callosum, basal ganglia, cerebellum, and the frontal lobe [21]. Deficits in brain structure due to PAE can further alter higher-order neurocognitive functions including intellectual functioning, executive control, language, memory and social cognition [22]. In comparison, evidence suggests that traumatic childhood experiences alter child brain development by dysregulating the Hypothalamic-Pituitary-Adrenal Axis (HPA-Axis), resulting in elevated basal cortisol levels [23–26]. This dysregulation of the HPA-Axis can further alter brain development and corresponding brain function [27]. Multiple studies have demonstrated HPA-axis dysregulation being associated with brain alterations in regions associated with neurocognitive function including the prefrontal cortex, amygdala, hippocampus [28–30]. The interactions between these three brain structures are further responsible for neurocognitive functioning, including memory and learning, which is relevant to the present discussion [31].

Given in-utero alcohol and/or postnatal complex trauma exposure can result in neurocognitive impairments, it is imperative to better understand the relative contribution of such deficits amongst pediatric patients who may have experienced both PAE and complex trauma. Recent studies have contributed to our understanding of the intersectionality of PAE and postnatal adversity [32,33]. The present study builds on these reviews by independently comparing the effects of PAE and complex trauma on 9 neurocognitive domains. CNS impairments including neurocognitive deficits are a hallmark of the FASD diagnosis, but since both PAE and recurrent post-natal trauma lead to neurodevelopmental impairments, a better understanding of this complex interplay is critical. Recent evidence has identified a disproportionately high percentage of FASD-diagnosed pediatric patients in correctional or care facilities, special education programs, and other specific populations [34].

Youth in such subpopulations may have increased ACE exposures which can further contribute to observed neurocognitive profiles. High rates of postnatal adversity have been seen in retrospective chart studies for children referred to FASD assessments, irrespective of their ultimate diagnosis [35]. Evaluating neurocognitive profiles through a trauma-informed lens can reshape how FASD is diagnosed, provide pediatric patients with targeted supports, and guide families into more effective treatments that focus on the root cause of neurocognitive impairments.

A preliminary search of MEDLINE, the Cochrane Database of Systematic Reviews and *JBI Evidence Synthesis* located no current or underway scoping or systematic reviews comparing the neurocognitive profiles of FASD and complex trauma were identified. A systematic review and meta-analysis published in 2023 highlighted neurocognitive outcomes of children with complex trauma but did not compare the deficits with FASD-diagnosed children [27]. This comparison is a novelty of the present study. Similarly, a paper in 2009 summated evidence to outline the neurocognitive profile of children with FASD but a comparison to complex trauma was not made [22]. Therefore, the objective of this review is specifically to compare the neurocognitive profiles of FASD and complex trauma in the pediatric population.

## Review question

How do the neurocognitive profiles of FASD and complex trauma compare in the pediatric population?

## Inclusion criteria

### Participants

The selected studies will be longitudinal, prospective or retrospective in design and will include participants with a clinical FASD diagnosis, participants who have experienced complex trauma without an FASD diagnosis, or participants who have both an FASD diagnosis along with complex trauma exposure. We will only be looking at studies where all participants were below the age of 19 as we are focusing on the pediatric population. Fetal alcohol spectrum disorder (FASD) is a diagnostic term used to describe the neurocognitive, developmental, and physical effects of PAE [6]. Complex trauma is defined as multiple and/or chronic, interpersonal traumatic events that occurred in childhood or adolescence [14–17]. To focus our research question, we will not include studies that only assessed the neurocognitive effects of prenatal alcohol exposure. We will not be including papers that evaluated the neurocognitive effects of a single, acute traumatic event.

### Concept

The concept of this scoping review is to compare the neurocognitive outcomes of FASD and complex trauma. The neurocognitive outcomes that we will assess include motor, cognition/intelligence quotient (IQ), language, academic achievement, memory, attention, executive functioning, and affect regulation [36]. Various FASD guidelines have described the domain of affect regulation to be considered impaired if there is a co-occurring mental health disorder including Major Depressive Disorder, Persistent Depressive Disorder, Disruptive Mood Dysregulation Disorder, Separation Anxiety Disorder, Selective Mutism, Social Anxiety Disorder, Panic Disorder, Agoraphobia, or Generalized Anxiety Disorder [6,36,37]. Other neurocognitive outcomes are measured by a standardized assessment tool or questionnaires that can be self or parent reported. These categories of neurocognitive outcomes will be narratively compared in children diagnosed with FASD to youth who have experienced complex trauma.

### Context

Racial and ethnic disparities amongst Black, Indigenous, and People of Color (BIPOC) exist with regards to FASD prevention, intervention, and diagnosis [38]. In addition, a large subset of FASD-diagnosed youth are exposed to correctional or care facilities and the effects of colonialism and intergenerational trauma continue to disproportionately affect Indigenous peoples, exacerbating health disparities [34,39]. The effects of intergenerational trauma can predispose Indigenous youth to greater amounts of ACEs as reported in a 2022 systematic review [40]. With the understanding that FASD diagnosis and ACEs are reported as disproportionately higher in some Indigenous and BIPOC populations, a better understanding of the cognitive profiles of associated with FASD and complex trauma respectively, could increase understanding of these health disparities and contribute to more equitable health outcomes.

### Types of sources

Observational studies including prospective and retrospective cohort studies, case-controlled studies and analytical cross-sectional studies will be considered for inclusion. Conference proceedings will be included if they report applicable data. We will consider published and unpublished studies including theses and dissertations as applicable. This scoping review will exclude experimental and quasi-experimental study designs, systematic reviews, and text and opinion papers.

## Methods

The proposed scoping review will be conducted in accordance with the JBI methodology for scoping reviews [41] with some modifications to the search and study selection process to ensure the feasibility of the review. Namely, we will search for self-identified systematic reviews, scoping reviews and meta-analyses that investigate the impact of FASD and/or complex trauma on neurocognitive outcomes. We will then retrieve the studies in each review and screen them for eligibility. We made these modifications due to an unmanageable number of results being retrieved by our initial test searches. We believe the high number is due to the lack of wide-spread use in the literature of the term “complex trauma,” which necessitates using broader terms in our search, such as “child abuse” and “adverse childhood experiences.” We believe that maintaining our review scope and inclusion criteria will deliver the most impactful and useful results and will report these modifications, and the resulting limitations to our review, transparently. This protocol is reported in accordance with the PRISMA-P guidelines (see S1 File) [42]. We estimate the record screening to be completed in 4 months, the data extraction to be completed in an additional 4 months, and the results to be available in 9 months. These stages have not currently been completed.

### Search strategy

A three-step search strategy will be utilized in this review. First, a preliminary limited search of MEDLINE (Ovid) and PsycINFO (EBSCO) was conducted to identify relevant articles to analyze the index terms and keywords contained in the titles, abstracts and author keywords. In addition, we reviewed the search strategies of relevant reviews and the studies included in these reviews were also analyzed for index terms and keywords. The located index terms and keywords used to describe the articles were employed to develop a full search strategy for MEDLINE (Ovid). The search strategy uses a search filter to retrieve pediatric studies [43] and a modified filter to retrieve systematic and scoping reviews [44,45] (see S2 File). We intend to locate both published and unpublished studies.

We will search MEDLINE (Ovid), Embase (Ovid), PsycINFO (EBSCO), Scopus and Google Scholar. The search strategy, including all identified keywords and index terms, will be adapted for each included database and/or information source. The reference list of all included sources of evidence included in the review will be screened for additional studies and we will use Scopus to search the citing studies.

We will not limit our search to English or by publication date. For non-English papers we will attempt to translate them using Google Translate. If a clear translation is not possible, the study will be excluded as we do not have the resources for a translator.

### Study/Source of evidence selection

Our study selection will be completed in two steps due to limiting our search to meta-analyses and systematic and scoping reviews. See Table 1 for a description of each step. First, following the search, all identified reviews will be collected and uploaded into Covidence (Veritas Health Innovation, Melbourne, Australia) and duplicates removed. We will conduct a pilot test to ensure concurrence among reviewers. Then, the reviews will be screened independently and in duplicate, first by title and abstract and then by full-text review. The reviewers will judge inclusion based on if one or more studies included in the review meets our inclusion criteria.

**Table 1:** Search and study selection steps.

Step	Description of Step
1. Locate relevant reviews	We will search for scoping and systematic reviews and meta-analyses. The results will be upload to Covidence and de-duplicated.
2. Assess eligibility of reviews	All reviews will be screen to determine if they include one more study that meets our inclusion criteria.
3. Extract studies	Included studies from the reviews will be extracted. All studies will be uploaded into Covidence and de-duplicated.
4. Assess eligibility of studies	All studies will be screened against the eligibility criteria.
5. Citation searching	We will conduct backward and forward citation searching of all included studies to locate any missing relevant studies.

Secondly, all studies included in the relevant reviews will be retrieved and uploaded into Covidence and duplicates removed. Following a pilot test, titles and abstracts will then be screened against the inclusion criteria independently and in duplicate. Potentially relevant full text will be retrieved and imported into Covidence and will be assessed thoroughly against the inclusion criteria independently and in duplicate.

At the full text stage, when a source does not meet the inclusion criteria, the reason will be recorded and reported in the scoping review. Any disagreements between the reviewers will be resolved through consensus, or by involving an additional reviewer. The results of the search and the study selection process will be reported in full in the final scoping review and presented in a PRISMA flow diagram [46].

### Data extraction

Data will be extracted from papers included in the review independently and in duplicate using a data extraction tool developed by the reviewers. Data will be extracted in Covidence and will include, at a minimum, details about the study participants demographics and the number of ACE exposures.

See S3 File for a draft data extraction tool, which will be modified and revised as necessary before beginning the data extraction of each included evidence source. Any modifications made to the tool will be reported in the scoping review. Any disagreements among the reviewers during data extraction will be resolved through consensus, or by involving an additional reviewer. When appropriate, we will contact the authors of included sources to request missing or additional data, where required. When we do not receive a response within 1 week, we will contact the authors one additional time.

### Data analysis and presentation

We envision the results of the scoping review to be present in a tabular format based on the variables outlined in the draft extraction form. These variables include the nine different neurocognitive outcomes mentioned in the draft extraction form and how they compare in FASD versus complex trauma exposure. A narrative summary will accompany the tabulated results and will describe how the results relate to the reviews objective and question(s). The narrative summaries will then be compared to determine how the neurocognitive profiles of FASD and complex trauma compare in the pediatric population. Graphic organizers will accompany these results to highlight information such as how many relevant papers we found pertaining to each neurocognitive outcome as well as the year in which these articles were published. This will help identify trends in the available literature.

## Supporting information

S1 File PRISMA-P checklist. (DOCX)

S2 File MEDLINE search strategy. (DOCX)

S3 File Draft data extraction instrument. (DOCX)
