# Supplementary material for: How do the neurocognitive profiles of FASD and complex trauma compare in the pediatric population?: A scoping review protocol
Source: PLoS One. 2025 Aug 5;20(8):e0328845. doi: 10.1371/journal.pone.0328845 (PMC12324080; doi:10.1371/journal.pone.0328845)
Supplement: S2 File — (DOCX) [file pone.0328845.s002.docx]

### MEDLINE Search strategy

**Database:**

Ovid MEDLINE(R) ALL <1946 to February 20, 2025>

| **#** | **Query** | **Results from 24 Feb 2025** |
| --- | --- | --- |
| 1 | Fetal Alcohol Spectrum Disorders/ | 4,847 |
| 2 | (f?etal alcohol or (prenatal* adj4 alcohol)).tw,kf. | 6,852 |
| 3 | 1 or 2 | 8,129 |
| 4 | Adverse Childhood Experiences/ | 4,731 |
| 5 | (adverse childhood experiences or early adverse experiences or early childhood adversity or early life adversity or childhood adversity).tw,kf. | 8,987 |
| 6 | Child Abuse/ or Child Abuse, Sexual/ or Domestic Violence/ or Psychosocial Deprivation/ or Intimate Partner Violence/ or Physical Abuse/ | 52,201 |
| 7 | (abuse or maltreatment or maltreated or neglect or neglected).tw,kf. | 229,500 |
| 8 | ((witness* or expos*) adj4 violence).tw,kf. | 7,341 |
| 9 | parental violence.tw,kf. | 256 |
| 10 | Stress Disorders, Post-Traumatic/ | 44,973 |
| 11 | (developmental trauma or complex trauma or Complex PTSD or complex post traumatic stress disorder or childhood trauma).tw,kf. | 7,808 |
| 12 | or/4-11 | 303,296 |
| 13 | psychomotor performance/ or motor skills/ | 94,432 |
| 14 | (gross motor or fine motor or motor skills or motor performance or graphomotor or visual-motor integration).tw,kf. | 30,746 |
| 15 | Cognition/ or Intelligence/ or Cognitive Dysfunction/ | 196,220 |
| 16 | (cognition or intelligence or intellectual abilit* or intellectual function* or processing speed).tw,kf. | 246,082 |
| 17 | (cognitive adj3 (outcome? or development or ability or function* or process* or impairment or disability or wellbeing or well-being or control or flexibility)).tw,kf. | 256,868 |
| 18 | Language Development/ | 12,804 |
| 19 | (language or speech or verbal or reading or spelling).tw,kf. | 535,561 |
| 20 | academic performance/ or academic success/ | 4,823 |
| 21 | ((academic or education*) adj2 (success or achievement or attainment or performance)).tw,kf. | 35,957 |
| 22 | (numeracy or number processing or mathematical ability or mathematical skill?).tw,kf. | 3,401 |
| 23 | memory/ or memory, episodic/ or memory, long-term/ or memory consolidation/ or memory, short-term/ or spatial memory/ | 114,908 |
| 24 | (learning or memory).tw,kf. | 868,884 |
| 25 | attention/ or attentional bias/ | 90,094 |
| 26 | (attention or attentional bias or information processing).tw,kf. | 660,787 |
| 27 | Executive Function/ | 21,716 |
| 28 | (executive function* or set shifting or task switching or inhibition or inhibitory control or problem solving or problem planning or concept formation or visual-spatial or visual construction or visual perception).tw,kf. | 1,151,611 |
| 29 | Inhibition, Psychological/ | 13,306 |
| 30 | Impulsive Behavior/ | 10,390 |
| 31 | (impulsiv* or impulse control).tw,kf. | 30,929 |
| 32 | hyperactivity.tw,kf. | 60,826 |
| 33 | Affective Symptoms/ | 14,229 |
| 34 | (affect regulation or affective symptoms or self-regulat*).tw,kf. | 22,463 |
| 35 | (regulat* adj3 impair*).tw,kf. | 5,969 |
| 36 | Depressive Disorder, Major/ | 42,093 |
| 37 | major depressive disorder.tw,kf. | 36,540 |
| 38 | Persistent Depressive Disorder.tw,kf. | 204 |
| 39 | disruptive mood dysregulation disorder.tw,kf. | 257 |
| 40 | Anxiety, Separation/ | 2,211 |
| 41 | Separation Anxiety.tw,kf. | 1,913 |
| 42 | Mutism/ | 1,195 |
| 43 | selective mutism.tw,kf. | 304 |
| 44 | Phobia, Social/ | 1,440 |
| 45 | (social anxiety disorder or social phobia).tw,kf. | 7,561 |
| 46 | Panic Disorder/ | 7,414 |
| 47 | (panic disorder? or panic attack?).tw,kf. | 12,686 |
| 48 | Agoraphobia/ | 2,696 |
| 49 | agoraphobia.tw,kf. | 3,409 |
| 50 | Generalized Anxiety Disorder.tw,kf. | 11,184 |
| 51 | Adaptation, Psychological/ | 108,281 |
| 52 | (adaptive behavio?r or adaptive functioning or daily living skills or communication).tw,kf. | 365,611 |
| 53 | social behavior/ or social skills/ | 62,853 |
| 54 | (social skills or social behavio?r or socio-emotional skills or socialization).tw,kf. | 32,804 |
| 55 | exp Epilepsy/ | 132,157 |
| 56 | (epilepsy or seizure?).tw,kf. | 229,610 |
| 57 | exp Neuroimaging/ | 205,423 |
| 58 | (neuroimaging or brain imaging or occipitofrontal circumference or neurophysiology or neuroanatomy or structural brain abnormal*).tw,kf. | 109,064 |
| 59 | Sensation Disorders/ | 5,444 |
| 60 | (sensory profile? or sensory disorder?).tw,kf. | 3,124 |
| 61 | Neuropsychological Tests/ | 107,333 |
| 62 | (neuropsychological or neuro psychological or neurocognitive or neuro cognitive or neurodevelopment* or neuro development*).tw,kf. | 151,591 |
| 63 | or/13-62 | 4,344,468 |
| 64 | 12 and 63 | 74,896 |
| 65 | 3 or 64 | 82,781 |
| 66 | exp Child/ or exp infant/ or adolescent/ or exp pediatrics/ or child, abandoned/ or exp child, exceptional/ or child, orphaned/ or child, unwanted/ or minors/ or (pediatric* or paediatric* or (child* not childbearing) or newborn* or congenital* or infan* or baby or babies or neonat* or pre-term or preterm* or premature birth* or NICU or preschool* or pre-school* or kindergarten* or kindergarden* or elementary school* or nursery school* or (day care* not adult*) or schoolchild* or toddler* or boy or boys or girl* or middle school* or pubescen* or juvenile* or teen* or youth* or high school* or adolesc* or pre-pubesc* or prepubesc*).mp. or (child* or adolesc* or pediat* or paediat*).jn. | 5,409,268 |
| 67 | 65 and 66 | 36,809 |
| 68 | "systematic review"/ | 284,105 |
| 69 | "Review Literature as Topic"/ | 8,877 |
| 70 | Systematic Reviews as Topic/ | 14,170 |
| 71 | Meta-Analysis as Topic/ | 25,511 |
| 72 | Meta-Analysis/ | 213,171 |
| 73 | (((systematic or state-of-the-art or scoping) adj2 (review* or overview* or assessment*)) or ((systematic or evidence) adj1 assess*) or "research evidence" or meta-analy* or metaanaly* or metasynthe* or meta-synthe*).ti,ab,kf. | 576,894 |
| 74 | (systematic review or meta-analysis).pt. | 371,619 |
| 75 | or/68-74 | 616,360 |
| 76 | 67 and 75 | 1,458 |
