# Supplementary material for: How do the neurocognitive profiles of FASD and complex trauma compare in the pediatric population?: A scoping review protocol
Source: PLoS One. 2025 Aug 5;20(8):e0328845. doi: 10.1371/journal.pone.0328845 (PMC12324080; doi:10.1371/journal.pone.0328845)
Supplement: S3 File — (DOCX) [file pone.0328845.s003.docx]

### Draft Data Extraction Instrument

| **DATA** | **VALUE** |
| --- | --- |
| Diagnosis/Exposure | 1. FASD 2. Complex Trauma |
| Neurocognitive Outcome | 1. Motor 2. Cognition/Intelligence Quotient (IQ) 3. Language 4. Academic Achievement 5. Memory 6. Attention 7. Executive Functioning 8. Memory 9. Affect Regulation |
| Age |  |
| Gender |  |
| Race |  |
| Socioeconomic Status |  |
| # of ACE exposures |  |
| Housing Exposure | 1. Foster Care 2. Adopted |
